# Ultrasonography: A novel approach to central venous cannulation

**DOI:** 10.4103/0972-5229.60174

**Published:** 2009

**Authors:** Ankit Agarwal, Dinesh K. Singh, Anil P. Singh

**Affiliations:** **From:** Department of Anaesthesiology and Intensive Care, BHU, Varanasi, India

**Keywords:** central venous cannulation, intensive care unit, ultrasound

## Abstract

**Background:**

Portable ultrasound machines are highly valuable in ICUs, where a patient's condition might not permit shifting the patient to the USG department for imaging. Traditionally central lines are put blindly using anatomical landmarks, which often result in complications such as difficulty in access, misplaced lines, pneumothorax, bleeding from inadvertent arterial punctures, etc. Ultrasonography provides “real time” imaging, i.e., the needle can be visualized entering the vein.

**Aims:**

We performed a study to compare USG guided central venous cannulation (CVC) and conventional anatomical landmark approach to CVC, in terms of ease of cannulation, time consumed, and associated complications.

**Settings and Design:**

The study was performed in a 16-bed open ICU. Eighty patients were randomly divided in two groups.

**Materials and Methods:**

The right internal jugular vein (IJV) was cannulated in all. In Group I, a portable ultrasound machine was used during cannulation. The vessels were visualized in the transverse section with the internal carotid artery (ICA) identified as a circular pulsatile structure, while the IJV as a lateral, oval nonpulsatile structure). The needle was inserted perpendicular to the skin under visualization on the US screen. Central venous line was then inserted by the Seldinger technique. In Group II, CVC was performed by the conventional landmark approach. The parameters studied included time for insertion, attempts required, and complications encountered.

**Statistical Analysis:**

The database of all parameters was analyzed using SPSS software version 10.5.

**Results:**

The mean time to successful insertion was 145 and 176.4 sec in groups I and II, respectively (*p* = 0.00). An average of 1.2 attempts per cannulation was required for group I, while 1.53 for group II (*p* = 0.03): 10% witnessed arterial puncture and 2.5% pneumothorax in group I and none in group II.

**Conclusion:**

USG-guided CVC is thus easier, quicker, and safer than landmark approach.

## Introduction

The availability of portable ultrasound machines has brought a revolution in the field of medicine. They are highly valuable in an Intensive care unit (ICU) setting, where the patient is so critical that he might not be able to be shifted to other departments for imaging, or his condition might not permit enough time for consultations from specialists. Portable ultrasound machines provide a point of care resource for the physicians, i.e., a facility is provided wherever a patient is.[1] Other uses of USG in ICU include assessment of cause of abdominal distention, DVT assessment, early identification of pericardial tamponade, identification of valvular pathology and left ventricular function, identification of structures in percutaneous tracheostomy, and assessment of pulmonary status also. A common use of ultrasonography in ICU is in central venous cannulation. Traditionally central lines have been put blindly using anatomical landmarks, which often resulted in complications such as arterial puncture, pneumothorax, hemothorax and air embolism. Ultrasonography provides “real time” imaging, i.e., the needle can be visualized entering the vein.[2,3] This reduces the chances of complications. Another advantage that USG cannulation offers is the visualization of vessels in hypotensive patients in whom carotid artery is difficult to palpate for landmark identification, for the IJV route CVC is required in ICU for the measurement of central venous pressure, administration of inotropes, parenteral nutrition, caustic drugs such as potassium, hemodialysis, cardiac pacing, in difficult peripheral venous access, etc. These are sometimes emergency procedures and USG CVC is reported to be faster than conventional approach. The “Stanford Evidence based practice center” has recommended USG CVC as one of the 11-point recommendations in “A critical analysis of patient safety practices.”[4] In some countries, CVC under US guidance is likely to be made compulsory in the near future.[5]

We performed a study to compare USG CVC and conventional landmark approach to CVC in terms of ease of cannulation, time consumed in the procedures, and associated complications.

## Materials and Methods

After approval from our hospital ethical committee, the study was performed in our 16-bed ICU catering for both medical and surgical patients. A total of 80 patients were included in the study, randomly allocated to 40 in each group. Right-sided internal jugular vein was cannulated in all the patients. All cannulations were done by either senior residents or consultants. All of them had undergone training in USG-guided cannulation techniques and had been performing the procedure for at least 1 year previously. Patients were placed in supine position and standard monitors were applied. A peripheral IV access was always ensured before starting the procedure. Part preparation and draping was done with aseptic precautions.

**Figure 1:**
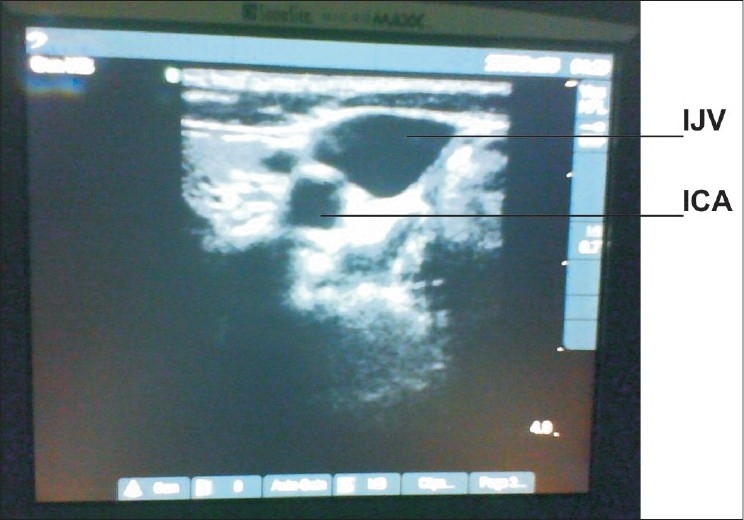
IJV and ICA as seen normally on scan (IJV = internal jugular vein, ICA = internal carotid artery)

**Figure 2:**
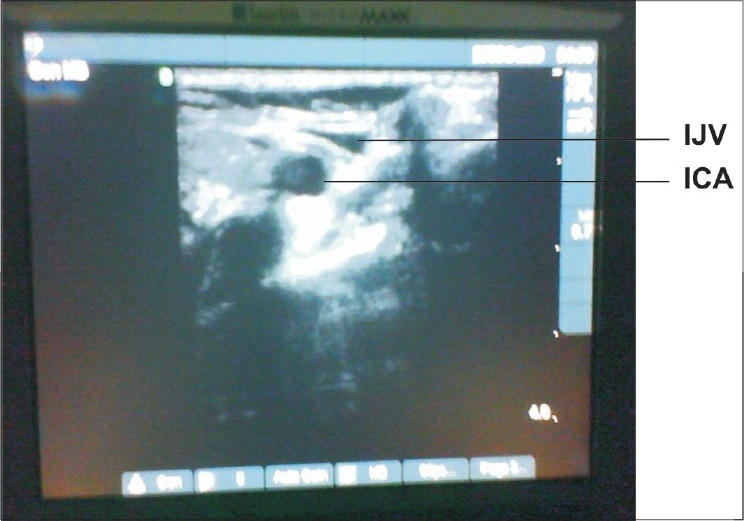
On applying pressure with US probe, IJV gets compressed while ICA remains as such

In Group I, a portable ultrasound machine “Sonosite Micromaxx®” with a 7.5-MHz probe was used. Sterile jelly was applied over the area to be scanned as well as the probe. The probe was then covered in a sterile sheath and placed over the triangle formed by the two heads of the sternocleidomastoid muscle (SCM). The vessels were visualized in the transverse section. Internal carotid artery was seen as a circular pulsatile entity, while the internal jugular vein was seen lateral to it as an oval nonpulsatile structure. On applying downward pressure with the ultrasound probe, the IJV got compressed whereas the ICA remained as such [Figures 1 and 2]. A blunt pointed probe was used to dimple the overlying skin, and indentation of the venous wall was seen and this served as the point for insertion of needle. The needle was then inserted directed perpendicular to the skin under visualization on the US screen. After successful aspiration of blood, a J-shaped guide wire was inserted through the hollow needle, and after single passage of a dilator through the guide wire, the central line was rail-roaded over the guide wire. The guide wire was withdrawn and all ports of the central line were checked for free flow of blood. After suturing, a transparent dressing was applied over the area of insertion.

In Group II, CVC was performed by the conventional landmark approach. The patient was placed supine with slight head down position and an IV fluid plastic bottle placed between the shoulder blades. The neck was turned slightly to the contralateral side and the apex of the triangle formed by the two SCMs was palpated for ICA pulsations. Once palpable, the ICA was pressed slightly medially with fingers of the left hand so that it does not overly the IJV. A heparinized central line needle was then inserted just lateral to the point of ICA pulsations, directed toward the ipsilateral nipple at an angle of 20°–30° with the skin. After successful aspiration of blood, rest of the procedure was similar to that in Group I.

After the procedure, a chest X-ray was routinely performed in all the patients to rule out a pneumothorax and to confirm the position of the central venous catheter tip.

In both the groups, the parameters studied included-

- Time (in seconds) from completion of skin prep and draping to successful aspiration of venous blood
- No. of attempts required for successful cannulation of IJV
- Complications encountered, if any

Exclusion criteria for the study included:

- Patients with previous CVC within 15 days
- Anatomical deformity, e.g., neck surgery, malignancy
- Burns on the site of insertion
- Emergency conditions not permitting time to arrange equipment for study
- Bleeding disorders

*Statistical Analysis* was done using SPSS software version 10.5.

## Results

The primary outcome, i.e., time required for insertion is summarized in Table 1. In group I, i.e., the USG group, the mean time to successful aspiration of venous blood after completion of draping was 145 secs, while in group II, i.e., the conventional group, this time was 176.43 secs. Statistically this difference was highly significant (*t* = 5.92, *p* = 0.00).

**Table 1:** Time from draping to successful aspiration of venous blood

	Mean (s)	±SD
Group I	145.00	16.98
Group II	176.43	23.48

Our next outcome measure was the no. of attempts required for successful cannulation [Table 2]. In Group I, i.e., the ultrasound group, five patients (12.5%) required more than one attempt, whereas in the conventional group 13 patients (32.5%) required more than one attempt. Statistically this difference was significant (*t* = 2.18, *p* = 0.03). An average of 1.2 attempts per cannulation was required for USG-guided cannulation, while for conventional method average 1.53 attempts were required.

**Table 2:** No. of attempts required for successful cannulation

	Group I	Group II
No. of patients requiring more than 1 attempt	5	13
Mean no. of attempts	1.20	1.53
±SD	0.48	0.68

We also tabulated the complications encountered in both the procedures [Table 3]. In the conventional group, we witnessed arterial puncture in four patients (10%), and pneumothorax in one patient (2.5%). There were no such complications in the ultrasound group.

**Table 3:** Complications encountered

	Group I	Group II
Arterial puncture	0	4
Pneumothorax	0	1

## Discussion

Complications during central venous catheterization (CVC) are not rare and can be serious. The use of ultrasound during CVC has been recommended to improve patient safety. The use of ultrasound during CVC is limited and is most strongly associated with the availability of the equipment.[6] Watters has philosophically summarized “Why only use a technology when things might be difficult, or when one has failed without it? I wear my seatbelt every time I drive a car, not just when the road is dangerous, or I've had a near miss.” He has appropriately quoted “There was an outcry when wearing seatbelts was made compulsory, but their adoption was a major advance in road safety.”[7] Similarly USG once started to be used generally is expected to be highly useful. The use of ultrasonography in experienced hands reduces the number of attempts and arterial punctures compared with the landmark method. After three or more attempts at insertion, mechanical complications increase by six times compared with a single attempt.[8] A disadvantage associated with USG-guided CVC, is procedure-related increased incidence of infection. But the use of a two-operator technique with sterile self-adhesive plastic and povidone iodine solution has reduced the incidence of infection.[9]

With our observations and results, we came to the conclusion that the USG approach took lesser time, required lesser attempts, and had lower incidence of complications for cannulation of the internal jugular vein. Regular use of USG for CVC will definitely benefit critically ill patients. It would be complimentary for any ICU to have portable USG facility, although a costly investment in a developing country like India, one must keep in mind that use of USG is a prudent approach as USG-guided CVC is easier, quicker, and safer than landmark approach.
